# Whole-Genome Sequencing Identified Co-Circulating VIM-Producing *Serratia bockelmannii* and *Serratia sarumanii* in a Neonatal Intensive Care Unit Cluster Investigation

**DOI:** 10.3390/pathogens15101033

**Published:** 2026-10-01

**Authors:** Giulia Bicchieraro, Alicia Yoke Wei Wong, Donatella Pietrella, Fabio Arena, Filippo Allegrucci, Stefania Troiani, Antonella Mencacci, Roberta Spaccapelo

**Affiliations:** 1 Department of Medicine and Surgery, University of Perugia, 06129 Perugia, Italy; giulia.bicchieraro@unipg.it (G.B.); aliciayokewei.wong@unipg.it (A.Y.W.W.); donatella.pietrella@unipg.it (D.P.); fabio.arena@unipg.it (F.A.); 2 Microbiology Unit, Santa Maria della Misericordia Hospital, 06129 Perugia, Italy; filippo.allegrucci@ospedale.perugia.it (F.A.); stefania.troiani@ospedale.perugia.it (S.T.)

**Keywords:** *Serratia marcescens* complex, *Serratia bockelmannii*, *Serratia sarumanii*, nosocomial outbreak, neonatal intensive care unit (NICU), carbapenemase-producing bacteria, *bla*_VIM-1_, whole-genome sequencing, antimicrobial resistance

## Abstract

*Serratia marcescens* complex bacteria are recognized causes of outbreaks in Neonatal Intensive Care Units (NICU). We investigated a cluster of VIM-producing *Serratia* spp. in the NICU of Santa Maria della Misericordia Hospital, Perugia, through a retrospective epidemiological and genomic analysis integrating clinical and surveillance data, antimicrobial susceptibility testing, environmental screening, and whole-genome sequencing (WGS). In November 2024, VIM-producing *Serratia* was isolated from eight neonates, of whom five developed clinical infections, including two bloodstream infections. Nine VIM-producing *Serratia* isolates recovered from these eight neonates and one non-VIM-producing isolate from a bloodstream infection (used for comparison) were analysed. Routine MALDI-TOF MS identified all isolates as *S. marcescens*, whereas WGS resolved them into two genomic lineages: seven *S. bockelmannii* ST388 isolates and three *S. sarumanii* ST382-like isolates. All VIM-producing *Serratia* carried *bla*_VIM-1_ and *bla*_SHV-12_ and showed multidrug-resistant phenotypes. Long-reads sequencing reconstructed complete, and almost identical, IncA-ST12 plasmids in all nine VIM-producing *Serratia* isolates; the plasmids carried *bla*_VIM-1_ and *bla*_SHV-12_ together with additional resistance determinants. Environmental, water, and healthcare-worker screening did not identify a *Serratia* reservoir. No new VIM-producing *Serratia* infections occurred after the November cluster. These findings show how WGS complements routine MALDI-TOF MS and rapid carbapenemase testing by providing the taxonomic, epidemiological, and plasmid-level resolution needed to investigate complex healthcare-associated outbreaks.

## 1. Introduction

Healthcare-associated infections remain a major concern in neonatal intensive care units (NICUs), where premature and critically ill neonates are particularly susceptible because of immune immaturity, prolonged hospitalization, invasive procedures, and frequent exposure to broad-spectrum antimicrobial agents [1,2,3]. Among Gram-negative bacteria, *Serratia* spp. are well-recognized causes of outbreaks in neonatal settings [2,4,5,6,7]. They may cause asymptomatic gastrointestinal colonization as well as conjunctivitis, pneumonia, bloodstream infection, sepsis, meningitis, and other invasive infections [8,9]. Colonization is of particular epidemiological importance because asymptomatic neonates may act as reservoirs for cross-transmission and may subsequently develop invasive disease. In a large outbreak in a Brazilian neonatal unit, 38 infants were colonized without clinical signs, whereas 15 developed clinical infection, including five cases of septicemia [10]. Other neonatal-unit investigations have likewise documented the simultaneous occurrence of colonization and invasive infection during *S. marcescens* outbreaks [11,12].

The epidemiology of these outbreaks may be complex. Although some episodes are sustained by a predominant clone, neonatal units may also experience the simultaneous or sequential circulation of genetically unrelated strains [13,14,15]. Three consecutive outbreaks caused by unrelated *S. marcescens* clones were documented in one NICU over a 35-month period [14], while a 10-year observational study from an Italian NICU identified several clusters involving multiple unrelated clones [16], making epidemiological reconstruction difficult when based only on routine microbiological data.

Transmission may occur directly between patients or indirectly through contaminated medical equipment, expressed breast milk, disinfectants, healthcare workers’ hands, or environmental reservoirs [4,17]. Water-associated environments, particularly sinks and drains, have repeatedly been implicated. In a longitudinal NICU investigation, *S. marcescens* was detected in an average of 44% of sampled sink drains, indicating that these sites may constitute persistent environmental reservoirs [18]. More recently, WGS linked a neonatal outbreak involving 12 affected infants to a predominant clone detected both in patients and in contaminated handwashing sinks [17]. However, an environmental source is not invariably identified [4,19]. In a Swiss neonatal-unit outbreak involving 20 colonized neonates, all 231 environmental samples were negative despite evidence of transmission between patients [20]. This makes it difficult to distinguish sustained patient-to-patient transmission from repeated acquisition from an unidentified reservoir. *Serratia* spp. may also persist in biofilms and show reduced susceptibility to some antiseptics and disinfectants, further complicating infection-control measures [21].

Antimicrobial resistance adds another level of complexity. Members of the genus possess intrinsic resistance mechanisms, including chromosomally encoded AmpC-type β-lactamases, and may additionally acquire extended-spectrum β-lactamases, including carbapenemases [22,23]. Antimicrobial susceptibility testing is essential for therapeutic decision-making, but resistance profiles alone provide limited epidemiological resolution. Unrelated isolates may display similar resistance patterns, whereas closely related isolates may differ because of the acquisition or loss of mobile resistance determinants [24].

Verona integron-encoded metallo-β-lactamases (VIM) are Ambler class B carbapenemases that hydrolyse a broad range of β-lactam, including carbapenems. Their activity is not inhibited by available inhibitors such as avibactam or vaborbactam [25], with aztreonam-avibactam being the combination with potential residual activity only. The genes encoding these enzymes, particularly *bla*_VIM-1_ and *bla*_VIM-2_, are commonly inserted within class 1 integrons and may be associated with transposons and conjugative plasmids [26]. These mobile genetic contexts facilitate dissemination both within bacterial lineages and between species occupying the same healthcare environment. The identification of *bla*_VIM_-positive isolates from several bacterial species may therefore reflect both clonal transmission and horizontal mobilization of mobile resistance elements. Interspecies clusters of *bla*_VIM-1_-positive *Enterobacterales* have been reported in Italian hospitals, including cases in which individual patients were simultaneously colonized by multiple VIM-producing species. In one such cluster, *bla*_VIM-1_, together with *bla*_SHV-12_ and other resistance determinants, was carried on a highly conjugative, broad-host-range IncA plasmid [27,28]. These observations are particularly relevant to outbreak investigations because identification of the same carbapenemase gene in different species does not, by itself, establish whether transmission involved independent strains or a shared mobile genetic element.

Accurate tracking of *Serratia* spp. outbreaks has also been complicated by recent taxonomic revisions within the *S. marcescens* complex. Isolates identified as *S. marcescens* by biochemical methods, 16S rRNA gene analysis, or mass spectrometry may belong to several closely related species, such as *Serratia sarumanii* and *Serratia bockelmannii* [29,30]. Recent genomic studies have confirmed that both species are represented among clinical isolates previously reported as *S. marcescens*, including isolates recovered during neonatal unit outbreaks [15,29,30]. Routine identification may therefore group together genetically distinct lineages and obscure the actual structure of an outbreak.

Whole-genome sequencing (WGS) has become an important tool for resolving the epidemiology of healthcare-associated *Serratia* outbreaks [31,32,33,34,35]. In neonatal settings, WGS has been used to separate apparently related isolates into distinct genomic clusters and to establish relationships between patient and environmental isolates that could not be established from susceptibility testing or conventional typing alone. In one NICU investigation, WGS resolved the outbreak into three genomic clusters and identified related isolates in drains and on a baby scale [31]. In other outbreaks, WGS supported links between patient isolates and contaminated sinks or confirmed close relatedness among patient isolates despite negative environmental cultures [17,20]. WGS can also refine taxonomic assignment and characterize resistance determinants, plasmid replicons, and other mobile genetic elements. Its role in outbreak investigation is therefore complementary to rapid molecular diagnostic assays: the latter provide timely detection of clinically relevant resistance genes. In this context, WGS is particularly informative when interpreted together with epidemiological data, since genomic relatedness alone cannot establish transmission pathways. The integration of patient timelines, ward organization, environmental findings, and genomic data can provide a more robust reconstruction of outbreak dynamics.

In November 2024, VIM-producing *Serratia* spp. emerged among neonates admitted to the NICU of Santa Maria della Misericordia Hospital, Perugia, causing both colonization and infection cases. We conducted a retrospective epidemiological and genomic investigation to reconstruct the cluster and determine whether it reflected transmission of a single *Serratia* lineage or a more complex pattern involving distinct genomic backgrounds and mobile resistance determinants. Clinical and surveillance data, environmental and healthcare-worker screening, antimicrobial susceptibility testing, and whole-genome sequencing were integrated to define the *Serratia* species and sequence types involved, characterize their resistance and plasmid profiles, assess the occurrence of *bla*_VIM_ in other Gram-negative species, and investigate a possible environmental contribution to transmission.

## 2. Materials and Methods

### 2.1. Study Design and Outbreak Investigation

Routine surveillance for rectal colonization by carbapenemase-producing Gram-negative bacteria is performed in the NICU at admission and every 7 days thereafter as part of institutional infection prevention and control initiatives. Clinical specimens are collected according to clinical indication, and isolates with phenotypic findings suggestive of carbapenemase production are subjected to molecular carbapenemase testing as part of routine diagnostic practice. Before November 2024, *Serratia* spp. had been detected in the unit, but no VIM-producing *Serratia* had been identified. On 6 November 2024, a VIM-producing *Serratia* isolate was recovered from an ocular infection in patient A. The identification of additional VIM-producing *Serratia* isolates in other neonates over the following days prompted an outbreak investigation. The present study was therefore conducted as a retrospective epidemiological and genomic investigation of this cluster, integrating patient-level surveillance data, clinical microbiology, environmental and healthcare-worker screening, antimicrobial susceptibility testing, and whole-genome sequencing (WGS).

The genomic analysis was performed retrospectively on isolates recovered during routine clinical, surveillance, and environmental investigations that had been stored and remained available in the laboratory collection. All available VIM-producing *Serratia* isolates from the November cluster were included. In addition, the only available non-VIM-producing *Serratia* bloodstream isolate from the same period was included as a comparator. Other VIM-producing Gram-negative clinical isolates recovered from neonates during the same investigation were also analysed to assess whether shared resistance determinants were circulating across different bacterial species within the same neonatal setting. Environmental isolates were selected among those available in the stored collection on the basis of their epidemiological relevance. The non-VIM-producing *Pseudomonas putida* isolate (Pp16) was included because a VIM-producing clinical *P. putida* isolate had been recovered from a neonate, whereas the VIM-producing *Pseudomonas oleovorans* isolate Po17 was selected to investigate the environmental occurrence and genomic context of *bla*_VIM_. Overall, 16 isolates were subjected to WGS: 10 clinical *Serratia* isolates, four additional clinical VIM-producing Gram-negative isolates, and two environmental *Pseudomonas* isolates.

The NICU comprises two open-space areas dedicated to intermediate and intensive care, containing 16 and 10 beds, respectively, together with two negative-pressure isolation rooms containing two beds each. Beds within the open-space areas are approximately 1.5 m apart. Detailed retrospective information on the exact relative position of individual neonates involved in the outbreak was not available. Approximately 26 neonates were cared for in the NICU each month during the study period. Between September and November 2024, 16 neonates tested positive for *Serratia*; eight of them carried VIM-producing *Serratia*, beginning with the first VIM-positive case identified on 6 November 2024.

Following recognition of the cluster, colonized or infected neonates were transferred to the isolation rooms when available or cohorted within a designated area of the NICU and remained under contact precautions until discharge. Dedicated nursing staff were assigned to the cohort during each shift. Contact precautions included the use of disposable gowns and gloves by healthcare workers and parents, together with hand hygiene performed in the adjacent anteroom before entering and after leaving the isolation area. Weekly microbiological surveillance was maintained throughout the outbreak investigation, together with additional environmental, water, and healthcare-worker hand sampling.

Between September and December 2024, a total of 340 clinical and surveillance specimens were screened for carbapenemase-producing Gram-negative bacteria: 23 in September, 19 in October, 133 in November, and 165 in December. The increase in the number of screened specimens during November and December reflected the intensified surveillance implemented following recognition of the cluster.

### 2.2. Clinical Specimen Processing and Bacterial Identification

Clinical specimens were cultured on MacConkey agar (BioMérieux, Brussels, Belgium) for the selective isolation of Gram-negative bacteria, including *S. marcescens*, and Columbia Agar with 5% Sheep Blood (COS) (BioMérieux, Brussels, Belgium). Plates were incubated aerobically at 37 °C for 24–48 h. Species identification was performed by matrix-assisted laser desorption/ionization time-of-flight mass spectrometry (MALDI-TOF MS; Bruker Daltonics GmbH & Co. KG, Bremen, Germany). All isolates selected for subsequent phenotypic and genomic analyses were stored at −80 °C until further processing.

### 2.3. Antimicrobial Susceptibility Testing and Carbapenemase Detection

Antimicrobial susceptibility testing was performed using the VITEK^®^ 2 automated system with AST-N438 card (BioMérieux, Marcy-l’Étoile, France) and broth microdilution with the Micronaut system (Merlin Diagnostika GmbH, Bornheim, Germany). Minimum inhibitory concentrations (MICs) were interpreted according to the clinical breakpoints established by the European Committee on Antimicrobial Susceptibility Testing (EUCAST) version 16.0. Carbapenemase production was initially assessed using the NG-Test^®^ CARBA 5 immunochromatographic assay, which detects and differentiates KPC-, NDM-, OXA-48-like-, VIM-, and IMP-type carbapenemases. This assay differentiates among KPC-, NDM-, OXA-48-like-, VIM-, and IMP-type carbapenemases. The presence of carbapenemase genes, including the Verona integron-encoded metallo-β-lactamase gene (*bla*_VIM_), was subsequently confirmed using the Xpert^®^ Carba-R molecular assay (Cepheid, Sunnyvale, CA, USA) and further investigated by whole-genome sequencing.

### 2.4. Environmental Investigation: High-Touch Surface Sampling and Water Sampling

High-touch surfaces were sampled in several areas of the NICU, including the intensive care room, intermediate care room, isolation rooms, and corridor. Sterile swabs were used to sample an area of approximately 100 cm^2^ at each site. The complete list of sampled surfaces is provided in Supplementary Table S1. Swabs were inoculated onto blood agar (BioMérieux, Brussels, Belgium) and CHROMID^®^ Carba chromogenic agar (BioMérieux, Brussels, Belgium) the for the recovery of total cultivable bacteria and carbapenemase-producing *Enterobacterales*, respectively. Plates were incubated aerobically at 37 °C for 48 h. Following incubation, colony-forming units were counted and recorded. Representative colonies growing on CHROMID^®^ Carba agar were identified by MALDI-TOF MS and screened for carbapenemase production using the phenotypic and molecular methods described above. Water samples were collected from sinks at different locations within the NICU. At each sampling point, 1 L of water was collected in a sterile polypropylene bottle containing sodium thiosulfate (Na_2_S_2_O_3_) at a final concentration of 18 mg/L to neutralize residual disinfectants and reduce the risk of false-negative culture results. Each 1-L water sample was filtered through a mixed cellulose ester membrane with a pore size of 0.45 µm and a diameter of 47 mm, using a sterile vacuum filtration apparatus. Following filtration, the membrane was aseptically transferred onto blood agar and CHROMID^®^ Carba chromogenic agar. Plates were incubated at 37 °C for 24–48 h. After incubation colonies were counted and representative isolates were identified by MALDI-TOF MS.

### 2.5. Genomic DNA Extraction and Whole-Genome Sequencing

For each isolate, a pure colony was inoculated into broth medium and cultured to obtain sufficient bacterial biomass. Genomic DNA was extracted using the QIAsymphony DSP Virus/Pathogen Kit (Qiagen, Hilden, Germany) in accordance with the manufacturer’s instructions. Sequencing libraries were prepared using the Illumina DNA Prep Kit with Nextera DNA CD Indexes (Illumina, San Diego, CA, USA). Library concentrations were measured using a Qubit™ Flex Fluorometer (Thermo Fisher Scientific, Waltham, MA, USA), while library fragment size distribution and overall quality were assessed using an Agilent 4150 TapeStation System with High Sensitivity DNA ScreenTape (Agilent Technologies, Santa Clara, CA, USA). Whole-genome sequencing was performed on an Illumina MiSeq platform using MiSeq Reagent Kit v3 (Illumina, San Diego, CA, USA) and MiSeq Reagent Kit v2 (Illumina, San Diego, CA, USA). Sequencing was conducted using a paired-end protocol generating 2 × 150-bp reads.

To resolve the genomic location of the major resistance determinants, all VIM-producing *Serratia* isolates (Sm1–Sm9) were additionally subjected to long-read sequencing using the GridION platform (Oxford Nanopore Technologies, Oxford, UK). Libraries were prepared with the Rapid Barcoding Kit 24 V14 (Oxford Nanopore Technologies, Oxford, UK) according to the manufacturer’s instructions. Long-read data were produced on R10.4.1 flowcells (ONT) and basecalled with GUPPY in High-accuracy mode.

### 2.6. Bioinformatic and Comparative Genomic Analyses

Raw paired-end sequencing reads in FASTQ format were first processed using the DRAGEN pipeline for quality control and read preprocessing. The resulting quality-filtered reads were subsequently analysed using tools available through the Galaxy Europe platform and services provided by the Center for Genomic Epidemiology (CGE). Species-level identification was performed using SpeciesFinder v4.0.4 [36], which compares sequencing reads against a curated database of complete genomes retrieved from the National Center for Biotechnology Information (NCBI) by K-mer alignment using KMA. De novo genome assembly was performed using SPAdes v3.15.5 [37] through Galaxy, and assembled genomes were annotated using Prokka v1.14.6 [38]. Pan-genome analysis was performed using Roary v3.13.0 [39]. The analysis included the study isolates and the following reference genomes: *S. marcescens* ELP1.10 (GenBank accession no. GCA_030291735.1), *S. marcescens subsp. marcescens Db11* (GCA_000513215.1), *Serratia sarumanii* strain K-M0228 (GCA_035749905.1), and *Serratia bockelmannii* strain K-L0361 (GCA_051804595.1). Genes present in at least 95% of the analysed genomes were defined as core-genome genes. Whole-genome average nucleotide identity (ANI) was calculated using FastANI v1.3 [40]. An ANI threshold of >95% was used to assign isolates to the same bacterial species. Phylogenetic relationships based on the core genome alignment were inferred using IQ-TREE v2.4.0 [41]. Genomic alignments and associated isolate metadata were explored using Phandango (https://jameshadfield.github.io/phandango/#/ accessed on 15 July 2026), and the final phylogenetic trees were visualized and annotated using the Interactive Tree of Life (iTOL) [42]. Plasmid replicons were identified using PlasmidFinder v2.1 [43], whereas plasmid mobility and predicted mobilization characteristics were assessed using MOB-Typer v3.1.9 [44]. Plasmid sequence types were assigned using the plasmid multilocus sequence typing approach implemented in the CGE pMLST platform [43,45]. Acquired antimicrobial resistance genes and resistance-associated mutations were investigated using ResFinder v4.6.0 [46], ABRicate v1.0.1 [47], and the Comprehensive Antibiotic Resistance Database (CARD) [48]. Virulence-associated genes were identified by ABRicate v1.0.1 [47] based on virulence factor database (VFDB) with 80% of coverage and 80% of identity. Multilocus sequence typing was performed using MLST v2.22.0 and the resulting sequence-type assignments were confirmed using the PubMLST database [49].

Nanopore long reads data analysis was performed using EPI2ME software v2.0.2. The raw data were assembled using FLYE v. 2.9.5-b1801, medaka v 2.2.0 was used to polish and improve the sequence accuracy of the assembled genome, and sequences were annotated by Bakta v 1.11.4. The obtained fasta files were visualised and mapped by Proksee tool (https://proksee.ca assessed on 15 July 2026) using CARD v4.0.1 as reference database for antibiotic resistance genes, GC content was assessed by GC content v1.0.2. Virulence-associated genes were identified by ABRicate v1.0.1 [47] based on virulence factor database (VFDB), to provide an additional cross-validation of the reported virulence-gene profiles.

### 2.7. Statistical Analysis

The statistical analysis was performed using GraphPad Prism software version 8.4.3 (GraphPad Software, San Diego, CA, USA). Differences in the number of accessory genes between the *S. bockelmannii* and *S. sarumanii* groups were assessed using the two-tailed Mann–Whitney U test. Statistical significance was defined as *p* < 0.05.

### 2.8. Ethics Statement

The study was conducted in accordance with the principles of the Declaration of Helsinki and applicable national regulations. The investigation was undertaken as part of routine infection prevention and control activities in response to a suspected healthcare-associated outbreak. Clinical and microbiological data were collected retrospectively and anonymized before analysis. No additional samples were collected, and no diagnostic or therapeutic interventions were performed specifically for research purposes. The study was approved by the Regional Ethics Committee—CER Umbria (approval no. 4457/23, approval date: 25 January 2023).

### 2.9. Availability of Data and Materials

The bacterial genome sequencing data generated and analysed during the present study have been deposited in the National Center for Biotechnology Information Sequence Read Archive (SRA) under BioProject accession n° PRJNA1498554.

## 3. Results

### 3.1. Epidemiological Characteristics and Outbreak Timeline

**Figure 1:**
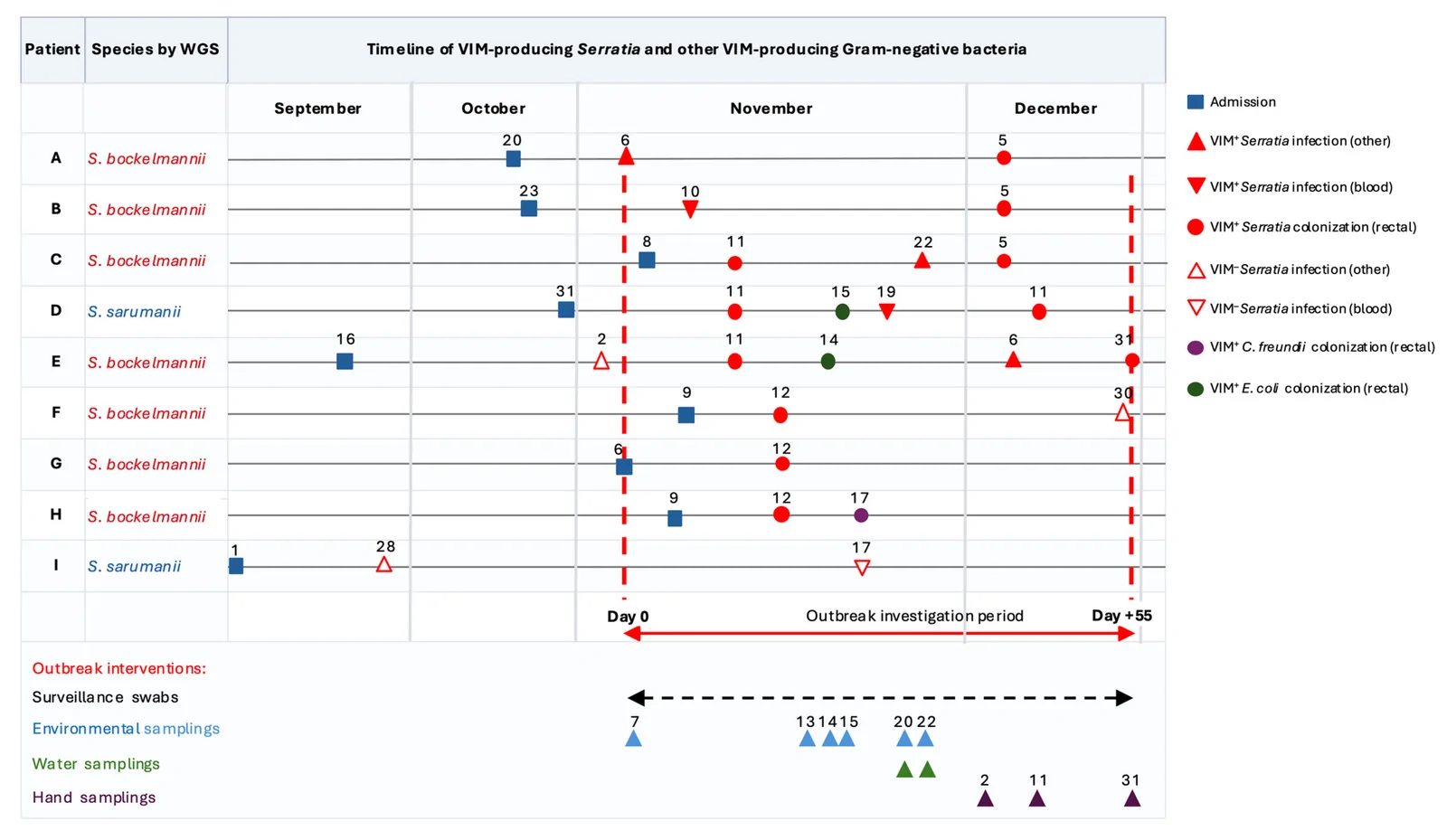
Timeline of the VIM-producing *Serratia* outbreak and associated outbreak investigation. Patient-level timeline showing admission, detection of VIM-producing (VIM^+^) and non-VIM-producing (VIM^−^) *Serratia*, and colonization by other VIM-producing Gram-negative bacteria between September and December 2024. The first VIM-producing *Serratia* infection, detected in patient A on 6 November 2024, was defined as Day 0 of the outbreak investigation. The lower panel indicates the timing of surveillance swabs (black dash arrow), environmental sampling, water sampling, and healthcare-worker hand sampling performed during the outbreak investigation (red dash lines). Follow-up extended from Day 0 to Day +55, corresponding to the last detection of VIM-producing *Serratia* in a previously affected neonate.

Between September and December 2024, 16 neonates admitted to the NICU tested positive for *S. marcescens* in clinical and/or surveillance specimens. The first VIM-producing *Serratia* was detected on 6 November 2024 in patient A, a female neonate born at 29 weeks of gestation who developed an ocular infection. This event was considered Day 0 of the outbreak investigation (Figure 1). Over the following six days, seven additional neonates (patients B-H) were identified as colonized or infected with VIM-producing *Serratia*, defining a cluster of eight affected patients. Five of the eight neonates developed clinically apparent infection during the observation period. Patient A presented with an ocular infection, patients C and E developed respiratory infections, and patients B and D developed bloodstream infections. Patient B, a female neonate born at 30 weeks of gestation, developed bloodstream infection 18 days after admission. Patient C, a male neonate born at 27 weeks of gestation, was first identified as colonized by rectal surveillance three days after admission and subsequently developed a respiratory infection eleven days later. Patient D, a female neonate born at 29 weeks of gestation, was initially identified as colonized by rectal surveillance 11 days after admission and developed bloodstream infection eight days later. Patient E, a female neonate born at 24 weeks of gestation, initially developed a respiratory infection caused by non-VIM-producing *Serratia* 47 days after admission. VIM-producing *Serratia* was subsequently detected by rectal surveillance nine days later, followed by a further respiratory infection caused by VIM-producing *Serratia*. Patients F, G and H were identified through rectal surveillance cultures within the first six days after admission. Patient I was a female neonate born at 24 weeks of gestation, developed respiratory infection 34 days after admission and later bloodstream infection/sepsis caused by non-VIM-producing *Serratia* 85 days after admission, during the November outbreak period. The demographic and clinical characteristics of the patients and *Serratia* isolates included in the phenotypic and genomic analysis are summarized in Table 1, while the temporal sequence of admissions, microbiological findings, and outbreak-control activities are shown in Figure 1.

Overall, ten *Serratia* isolates were included in the phenotypic and genomic analyses: nine VIM-producing isolates (Sm1-Sm9) recovered from eight neonates and one non-VIM-producing bloodstream isolate (Sm10) included as a comparator. The genomic investigation was retrospective and was performed on isolates recovered during routine clinical and surveillance activities that had been stored and remained available for analysis. All available VIM-producing *Serratia* isolates associated with the November cluster were included.

Following recognition of the VIM-producing *Serratia* cluster, colonized or infected neonates were transferred to the isolation rooms when available or cohorted within a designated area of the NICU and remained under contact precautions until discharge. Dedicated nursing staff were assigned to the cohort during each shift. Healthcare workers and parents were required to wear disposable gowns and gloves, and hand hygiene was performed in the adjacent anteroom before entering and after leaving the isolation area.

Weekly microbiological surveillance continued throughout the outbreak investigation, together with additional environmental, water, and healthcare-worker hand sampling performed as part of the outbreak investigation (Figure 1). During December, VIM-producing *Serratia* continued to be detected in surveillance cultures from neonates already involved in the November cluster, but no new neonates with VIM-producing *Serratia* were identified. The last positive VIM-producing *Serratia* surveillance culture was recorded on 31 December 2024 (Day +55) in a previously affected neonate. No subsequent neonatal specimens yielded VIM-producing *Serratia*. Thus, although carriage persisted in previously affected patients through December, no additional VIM-producing *Serratia* cases were identified after the November cluster. None of the affected neonates died.

Additional VIM-producing Gram-negative bacteria were recovered during the same investigation. These included two *E. coli* isolates one from patient D and one from patient E (Ec14 and Ec12 respectevely), *C. freundii* from patient H (Cf13), and *Pseudomonas putida* from neonate L (Pp15). Patient L was a female neonate who developed an ocular infection caused by non-VIM-producing *Serratia* 52 days after admission. A rectal swab collected 69 days after admission was positive for VIM-producing *P. putida* (Table 1). These isolates were included in the genomic investigation to examine the occurrence of *bla*_VIM_ among different Gram-negative species circulating in the same neonatal setting (Figure 1 and Table 1).

The study therefore aimed to reconstruct the cluster using epidemiological, phenotypic, environmental, and genomic data. The genomic analysis was performed retrospectively on isolates that had been recovered during the outbreak as part of routine clinical, surveillance, and environmental investigations, as described above, and were still available in the laboratory collection. Anyway, all available VIM-producing *Serratia* isolates from the November cluster were included. In addition, the only one available non-VIM-producing *Serratia* bloodstream isolate from the same period was selected as a comparator. Other VIM-producing Gram-negative clinical isolates recovered from the same neonatal setting were also included to investigate the possible circulation of shared resistance determinants. Environmental isolates were selected from those available in the stored collection on the basis of their epidemiological relevance.

### 3.2. Environmental Investigation

Environmental sampling was initiated on 7 of November, the day after identification of the first VIM-producing *Serratia* case, and repeated on 13, 14, 15, 20, and 22 November to cover the clinical areas occupied by the affected neonates (Figure 1). The NICU has a total capacity of 26 beds and comprises two open-space areas, dedicated to intermediate and intensive care, with 16 and 10 beds, respectively, together with two negative-pressure isolation rooms containing two beds each. Beds within the open-space areas are approximately 1.5 m apart. Precise retrospective information on the relative position of individual affected neonates within these areas was not available. During November 2024, the NICU operated at full occupancy therefore 26 neonates were present, including the eight neonates carrying VIM-producing *Serratia*. Following identification of the cluster, affected neonates were transferred to the isolation rooms when possible or cohorted within a designated area of the open-space NICU and remained there until discharge. A total of 56 samples were collected from high-touch surfaces during November; the complete list sampling sites is provided in Supplementary Table S1.

Water sampling was performed on 20 and 22 November and comprised 14 samples obtained from seven taps located in the intermediate-care, intensive-care, and isolation areas. Both filtered and unfiltered taps were sampled, including washbasins and designated water points; the complete list is provided in Supplementary Table S2. Healthcare-worker screening involved 60 staff members, including physicians, nurses, and healthcare assistants. Hand samples were collected before and after use of an alcohol-based hand rub. No *Serratia* isolates were recovered from surface, water, or healthcare-worker hand samples.

Several other bacterial species were recovered from high-touch surfaces, including *Pseudomonas mosselii*, *Pseudomonas putida*, *Klebsiella pneumoniae*, *Enterobacter asburiae*, *Pseudomonas aeruginosa*, *Pseudomonas oleovorans*, *Stenotrophomonas maltophilia*, *Enterococcus faecium*, and *Escherichia coli*. The environmental *E. faecium* isolate showed a vancomycin-resistant phenotype. Bacteria species recovered from water-associated samples included *Acinetobacter bereziniae*, *Acinetobacter junii*, *Pseudomonas mendocina*, *Pseudomonas alcaliphila*, *Pseudomonas aeruginosa*, and *Stenotrophomonas maltophilia*. In addition, a VIM-producing *P. oleovorans* isolate was also recovered from a sink trap.

Two environmental *Pseudomonas* isolates were selected for WGS on the basis of their epidemiological relevance. The non-VIM-producing *P. putida* (Pp16) was included because a VIM-producing clinical *P. putida* isolate (Pp15), had been recovered from patient L, allowing assessment of possible clinical-environmental relatedness. The VIM-producing *P. oleovorans* isolate (Po17) was selected to characterize and compare its *bla*_VIM_ determinant with those detected in the clinical isolates. These isolates represented the stored environmental samples considered most informative for evaluating a possible environmental contribution to the circulation of VIM-producing organisms. All environmental isolates were identified to species level by MALDI-TOF mass spectrometry.

### 3.3. Phenotypic Antimicrobial Susceptibility Testing of Clinical and Environmental Isolates

**Figure 2:**
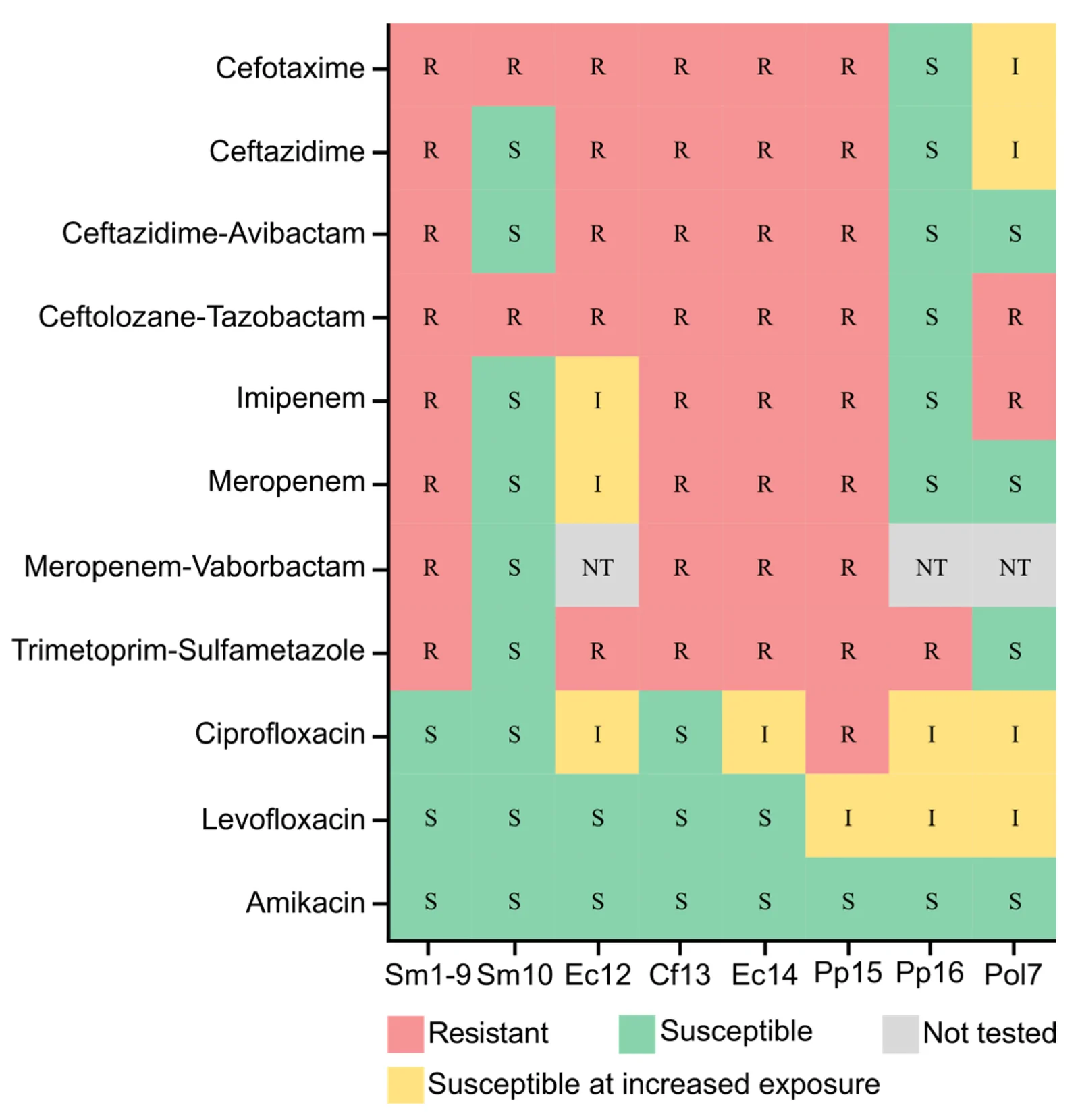
Phenotypic antimicrobial susceptibility profiles of clinical and environmental isolates. Heatmap of antimicrobial susceptibility profile of the VIM-producing *Serratia* isolates Sm1-Sm9, the non-VIM-producing *Serratia* isolate Sm10, four additional clinical Gram-negative bacteria, and two environmental *Pseudomonas* isolates. Red indicates resistant (R), green indicates susceptible (S), yellow indicates susceptible at increased exposure (I), and grey indicates not tested (NT).

The nine VIM-producing *Serratia* isolates showed similar multidrug-resistant phenotype, with resistance to most of antimicrobial agents tested (Figure 2). Resistance was observed to all tested cephalosporins and β-lactam/β-lactamase inhibitor combinations, including cefotaxime, ceftazidime, ceftazidime-avibactam, and ceftolozane-tazobactam. Resistance was also observed to all carbapenems tested, including imipenem, meropenem and meropenem-vaborbactam (Supplementary Table S3). Trimethoprim-sulfamethoxazole resistance was also observed in all VIM-producing *Serratia* isolates. By contrast, fluoroquinolones (ciprofloxacin and levofloxacin) and aminoglycoside amikacin retained in vitro activity against all nine VIM-producing *Serratia* isolates (Figure 2).

The non-VIM-producing *Serratia* isolate Sm10 showed a different susceptibility profile. The isolate was resistant to cefotaxime and ceftolozane-tazobactam but remained susceptible to all the other antibiotics tested. Consistent with the absence of *bla*_VIM_, carbapenem MICs for Sm10 remained below the EUCAST resistance breakpoints (Figure 2).

Antimicrobial susceptibility testing was also performed on four clinically relevant VIM-producing Gram-negative bacteria isolates recovered from rectal specimens. The two *E. coli*, Ec12 and Ec14, *C. freundii* Cf13, and *P. putida* Pp15 isolates exhibited multidrug-resistant phenotypes. All four isolates were resistant to the cephalosporins and β-lactam/β-lactamase inhibitor combinations tested. Carbapenem resistance was observed in Cf13, Ec14, and Pp15, whereas Ec12 was categorized as susceptible at increased exposure to both imipenem and meropenem. All four isolates were resistant to trimethoprim-sulfamethoxazole. In addition, *P. putida* Pp15 was resistant to ciprofloxacin and categorized as susceptible at increased exposure to levofloxacin (Figure 2).

The two environmental *Pseudomonas* spp. isolates had clearly different susceptibility profiles. The non-VIM-producing *P. putida* isolate Pp16 was susceptible to all β-lactam agents tested but resistant to trimethoprim-sulfamethoxazole; both fluoroquinolones were categorized as susceptible at increased exposure. In contrast, the VIM-producing *P. oleovorans* isolate Po17 was resistant to ceftolozane-tazobactam and imipenem, categorized as susceptible at increased exposure to the third-generation cephalosporins and fluoroquinolones tested. It remained susceptible to ceftazidime-avibactam, meropenem, trimethoprim-sulfamethoxazole, and amikacin (Figure 2).

### 3.4. Whole-Genome Sequencing and Genomic Characterization

Whole-genome sequencing was performed on ten clinical isolates identified by MALDI-TOF MS as *Serratia marcescens* and on six additional non-*Serratia* isolates recovered from clinical and environmental samples. Detailed sequencing output and assembly quality metrics, including raw and quality-filtered read counts, are reported in Supplementary Table S4.

De novo assembly of the ten *Serratia* genomes yielded a median genomes size of 5.3 Mb. Species-level genomic analysis resolved the isolates into two groups within the *S. marcescens* complex. Isolates Sm1, Sm2, Sm3, Sm5, Sm6, Sm7, and Sm8 were assigned to *Serratia bockelmannii*, whereas isolates Sm4, Sm9, and Sm10 were assigned to *Serratia sarumanii*. Thus, isolates identified as *S. marcescens* by routine methods comprised two distinct genomic lineages. This classification was further supported by multilocus sequence typing and core-genome phylogenetic analysis. For the six non-*Serratia* isolates, genomic species identification was concordant with MALDI-TOF MS. These comprised the clinical isolates *E. coli* (Ec12 and Ec14*), C. freundii* (Cf13) and *P. putida* (Pp15), together with the environmental isolates *P. putida* (Pp16) and *P. oleovorans* (Po17).

To further characterize the VIM-producing *Serratia* isolates, all nine isolates (Sm1–Sm9) were additionally subjected to Oxford Nanopore long-read sequencing. Detailed sequencing and assembly metrics, including raw and quality-filtered read counts, are reported in Supplementary Table S5. Long-read sequencing was concordant with the Illumina-based taxonomic assignments, confirming the presence of the two *Serratia* species identified within the cluster. In addition, long-read assemblies enabled reconstruction of complete circular plasmid sequences, allowing a more detailed characterization of their genomic structure. Long-read sequencing was not performed for the non-*Serratia* isolates.

### 3.5. Distribution of Antimicrobial Resistance Determinants

**Figure 3:**
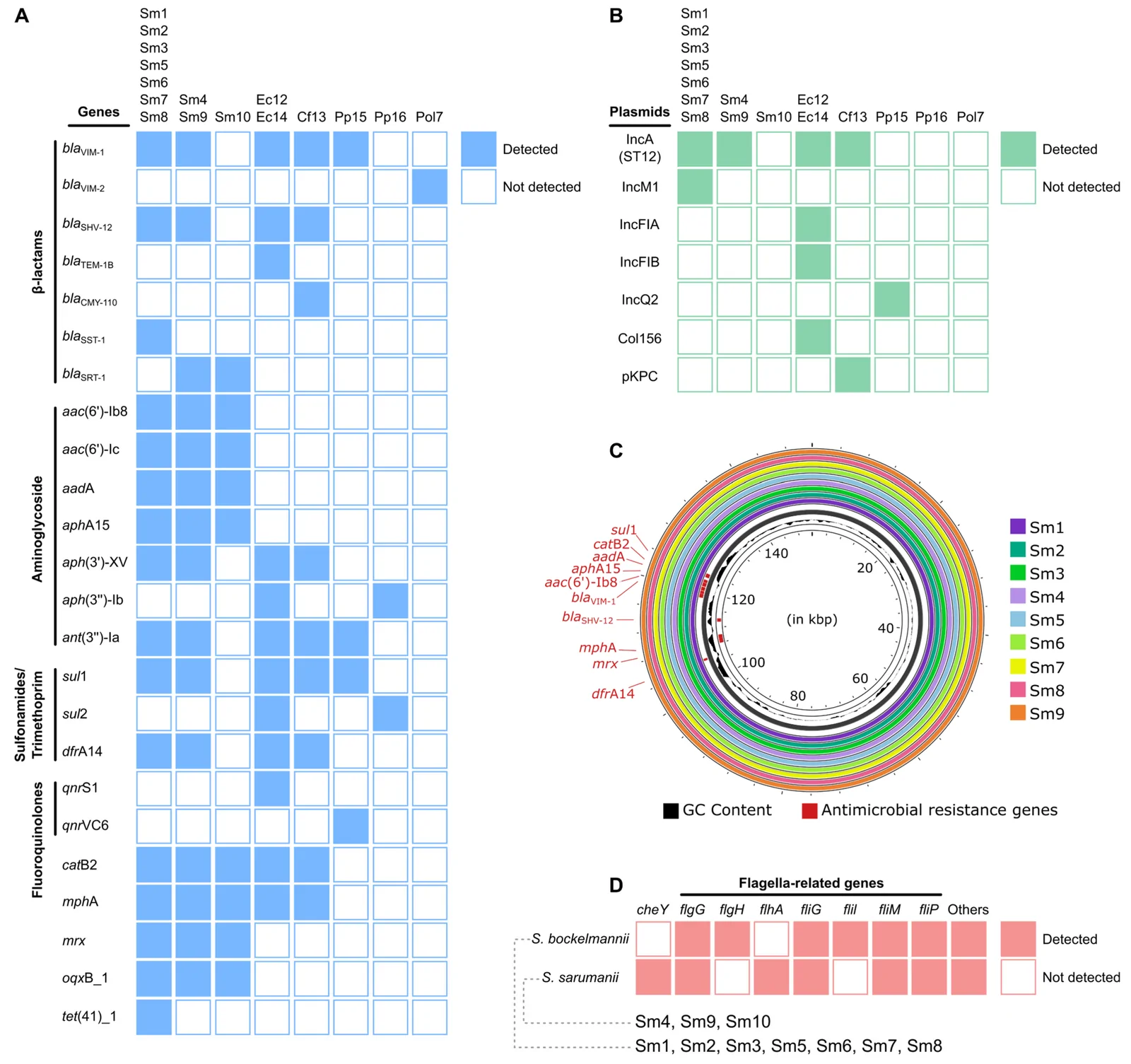
Distribution of antimicrobial resistance genes, plasmid replicons and virulence-associated genes among clinical and environmental isolates. (**A**) Heatmap showing the presence or absence of acquired and intrinsic antimicrobial resistance determinants identified by whole-genome sequencing. Isolates are arranged according to bacterial species and clinical or environmental origin. (**B**) Heatmap showing the presence or absence of plasmid replicon families identified using PlasmidFinder. (**C**) Heatmap showing the presence or absence of selected virulence-associated genes in the ten clinical *Serratia* isolates. (**D**) Reconstruction of IncA plasmid detected in 9 *Serratia* isolates Sm1-Sm9. Antibiotic resistance genes detected in the plasmids are in red, GC content in black.

Illumina short-read WGS data were analysed to characterize the antimicrobial resistance determinants of the clinical and environmental isolates (Figure 3A). All nine VIM-producing *Serratia* isolates (Sm1-Sm9) carried the metallo-β-lactamase gene *bla_VIM-1_* and the extended-spectrum β-lactamase gene *bla*_SHV-12_. Neither *bla*_VIM-1_ nor *bla*_SHV-12_ was identified in the non-VIM-producing isolate Sm10. Moreover, the distribution of intrinsic β-lactamase genes was consistent with species assignment: *bla*_SST-1_ was detected in the *S. bockelmannii* isolates Sm1, Sm2, Sm3, Sm5, Sm6, Sm7, and Sm8, while the *S. sarumanii* isolates Sm4, Sm9, and Sm10 carried *bla*_SRT-1_, encoding an inducible AmpC-type β-lactamase. The *Serratia* isolates also carried several aminoglycoside resistance determinants. Both *aac*(6′)-Ib8 and *aac*(6′)-Ic, together with *aad*A and *aph*A15, were detected across the *Serratia* isolates. The VIM-producing isolates additionally carried *aph*(3′)-XV and *ant*(3″)-Ia, which were absent from Sm10. The sulfonamide- and trimethoprim-resistance genes *sul1* and *dfrA14* were likewise detected in the VIM-producing *Serratia* isolates but not in Sm10. Additional resistance-associated genes identified among the *Serratia* isolates included *cat*B2, *mph*A, *mrx*, and *oqx*B_1. The tetracycline resistance gene *tet*(41)_1 was detected in the *S. bockelmannii* group but not in the *S. sarumanii* isolates. Overall, the VIM-producing isolates showed a broader acquired resistance-gene repertoire than the non-VIM-producing Sm10 isolate, in agreement with their multidrug-resistant phenotypes.

The two VIM-producing *E. coli* isolates, recovered from patients E and D (Ec12 and Ec14 respectively), were assigned to sequence type (ST) 12 and showed a broad range of acquired resistance genes. Both isolates carried *bla*_VIM-1_, *bl*a_SHV-12_, and *bla*_TEM-1B_, together with the aminoglycoside-resistance determinants *aph*(3′)-XV, *aph*(3″)-Ib, and *ant*(3″)-Ia. The genes *sul*1, *sul*2, and *dfr*A14 were also detected, supporting resistance to sulfonamides and trimethoprim. Both isolates additionally carried the plasmid-mediated quinolone-resistance gene *qnr*S1, which may contribute to reduced fluoroquinolone susceptibility and also harboured *cat*B2 and *mph*A (Figure 3A).

The *C. freundii* isolate Cf13 carried *bla*_VIM-1_, *bl*a_SHV-12_, and *bl*a_CYM-110_, consistent with its broad β-lactam resistance phenotype. The isolate also harbored the aminoglycoside-resistance genes *aph*(3′)-XV and *ant*(3″)-Ia, together with *sul*1 and *dfr*A14. The clinical *P. putida* isolate Pp15 carried *bla*_VIM-1,_ *ant*(3″)-Ia, and *sul*1. The additional genes *cat*B2 and *mph*A were also detected. The clinical *P. putida* isolate Pp15 carried *bla*_VIM-1_, *ant*(3″)-Ia, and *sul*1. It was also the only isolate in which *qnr*VC6 was detected (Figure 3A).

The two environmental *Pseudomonas* isolates exhibited distinct resistance gene profiles. The non-VIM-producing *P. putida* (Pp16) carried only *aph*(3″)-Ib and *sul*2 and lacked, as expected, VIM-type metallo-β-lactamase genes. By contrast, *P. oleovorans* (Po17) carried *bla*_VIM-2_. This distinction could not be resolved by the routine molecular carbapenemase assay, which identifies VIM-type carbapenemases without assigning the specific VIM allele, and was established by WGS. The *bla*_VIM-2_ allele in Po17 differed from the *bla*_VIM-1_ allele detected in the clinical *Serratia*, *E. coli*, *C. freundii*, and *P. putida* isolates, arguing against Po17 as the source of the *bla*_VIM-1_-positive clinical cluster.

Overall, the resistance profiles detected by WGS were broadly consistent with the antimicrobial susceptibility results. They differentiated the non-VIM-producing Sm10 isolate from the VIM-producing *Serratia* isolates and separated the *bla*_VIM-2_-positive environmental Po17 isolate from the *bla*_VIM-1_ positive clinical isolates.

### 3.6. Plasmid Replicon Content and Mobility Typing

Plasmid replicons were detected in 13 of the 16 sequenced isolates analysed by Illumina short-read sequencing. The detected replicon families included IncA, IncM1, IncFIA, IncFIB, IncQ2, Col156, and a pKPC-associated replicon (Figure 3B). An IncA replicon was detected in all VIM-producing *Serratia* isolates, as well as in *E. coli* Ec12 and Ec14 and *C. freundii* Cf13. In the *Serratia* and *E. coli* isolates, the IncA replicon showed 100% sequence coverage and 98.3% nucleotide identity to the corresponding reference sequence. Plasmid multilocus sequence typing assigned these replicons to plasmid sequence type 12 (pST12), based on the allelic profile A053-3, *parA*-7, *parB*-8, and *repA*-6. Mobility analysis classified them within the MOBH relaxase group. The IncA replicon detected in Cf13 showed 100% sequence coverage and 100% nucleotide identity to the reference sequence.

An IncM1 replicon was identified in all the *S. bockelmannii* isolates but was absent from the *S. sarumani* isolates (Figure 3B). IncM1 showed 100% sequence coverage and 99.5% nucleotide identity to the reference sequence and was assigned to the MOBP relaxase group. IncFIA, IncFIB, and Col156 replicons were detected in both *E. coli* isolates, with 100% sequence coverage and nucleotide identities of 99.7%, 99.4%, and 98.7%, respectively. Col156 was also assigned to the MOBP relaxase group. A pKPC-associated replicon was identified exclusively in *C. freundii* Cf13, with 100% sequence coverage and 100% nucleotide identity to the reference sequence. IncQ2 was detected only in the environmental *P. putida* isolate Pp16 (Figure 3B).

No plasmid replicons were detected in the clinical *P. putida* isolate Pp15 or in the environmental *P. oleovorans* isolates Po17 using the applied short-read in silico methods. Nevertheless, in Pp15, *bla*_VIM-1_ was identified on an assembled contig, in agreement with the positive Xpert^®^ Carba-R result (Figure 3A), but short-read sequencing did not allow its chromosomal or plasmid location to be resolved. By contrast, *bla*_VIM-2_ was chromosomally located in *P. oleovorans* Po17, indicating that this resistance determinant was not plasmid mediated in the environmental isolate.

Oxford Nanopore long-read sequencing enabled reconstruction of circular IncA-ST12 plasmids in all nine VIM-producing *Serratia* isolates. The reconstructed plasmids had an average length of approximately 151.7 kb. Pairwise comparison of the complete plasmid sequences showed 100% sequence coverage and 100% nucleotide identity across Sm1-Sm9, demonstrating that the IncA-ST12 plasmids were identical at the nucleotide sequence level. Long-read data directly demonstrated that *bla*_VIM-1_ and *bla*_SHV-12_ were located on the IncA-ST12 plasmid backbone. The same resistance region also contained *sul1*, *dfrA14*, *mphA*, *mrx*, *catB2*, *aadA*, *aphA15*, and *aac*(6′)-Ib8. Multiple *tra* genes involved in conjugative transfer were also present. Comparative plasmid mapping therefore supports the circulation of the same IncA-ST12 plasmid among the VIM-producing *Serratia* isolates. Long-read sequencing was not performed for the *E. coli* or *C. freundii* isolates. For these isolates, plasmid replicon typing and resistance-gene detection were based on Illumina short-read data; consequently, physical co-localization of *bla*_VIM-1_, *bla*_SHV-12_, and the IncA-ST12 replicon could not be directly demonstrated.

### 3.7. Virulence Genes

The distribution of virulence-associated genes was examined in the ten clinical *Serratia* isolates (Figure 3D). Both *S. bockelmannii* and *S. sarumanii* harboured a largely conserved repertoire of genes associated with flagellar assembly, motility, and chemotaxis, with no evidence of a distinctive high-virulence gene profile. The chemotaxis response regulator gene *cheY* and the flagellar biosynthesis gene *flhA* were detected in the *S. sarumanii* isolates but were absent from the *S. bockelmannii* isolates. Conversely, *flgH* and *fliF*, which encode components of the flagellar basal body, were detected in *S. bockelmannii* but were absent from *S. sarumanii*. The remaining flagella-associated genes shown in Figure 3C, including *flgG*, *fliG*, *fliU*, and *flgP*, were conserved across both lineages. Additional virulence-associated determinants, grouped under the category “Others” in Figure 3D, included *acrB*, *gndA*, *rcsB*, *rpoS*, and *tssC1*; all were detected in both lineages. Overall, the virulence-associated gene profiles were broadly conserved between the two species, while the differential distribution of *cheY*, *flhA*, *flgH*, and *fliF* provided an additional genomic feature distinguishing *S. bockelmannii* from *S. sarumanii*.

### 3.8. Comparative Genomic Analysis and Population Structure of the Serratia Isolates

**Figure 4:**
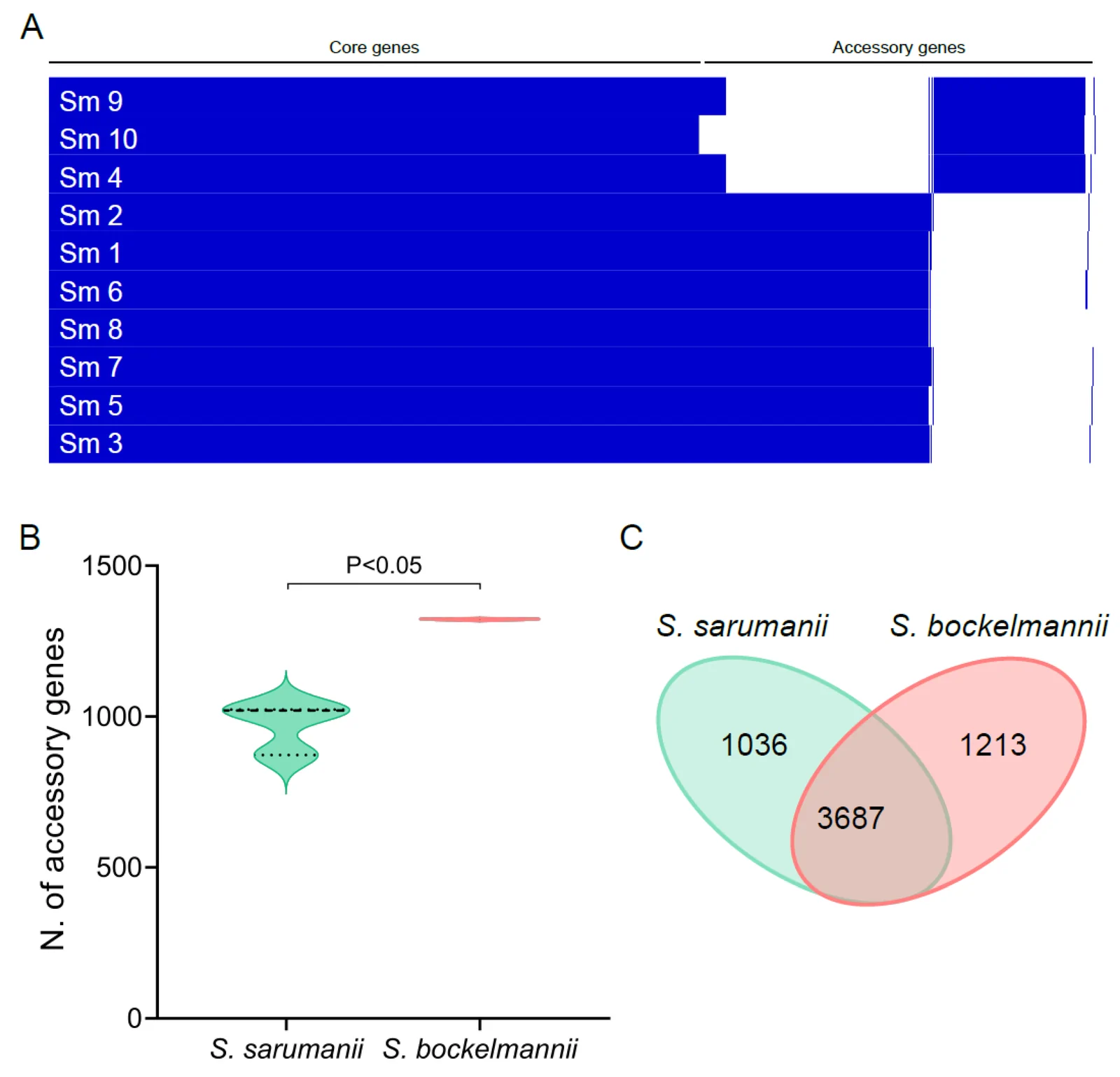
Pan-genome analysis of the ten clinical *Serratia* isolates. (**A**) Gene presence-absence matrix showing the distribution of core and accessory genes among the isolates. Blue indicates gene presence and white indicates gene absence. (**B**) Distribution of accessory gene counts in *S. sarumanii* and *S. bockelmannii*. *p* < 0.05. (**C**) Venn diagram showing the genes shared between the two lineages and those associated exclusively with either the *S. sarumanii* or *S. bockelmannii* group.

Pan-genome analysis of the ten clinical *Serratia* isolates shown two distinct gene-content profiles corresponding to the species assignments obtained by whole-genome sequencing. The *S. sarumanii* isolates Sm4, Sm9, and Sm10 clustered separately from the *S. bockelmannii* isolates Sm1, Sm2, Sm3, Sm5, Sm6, Sm7, and Sm8 in the gene presence-absence matrix (Figure 4A). The *S. bockelmannii* isolates carried a significantly higher number of accessory genes than the *S. sarumanii* isolates (*p* < 0.05; Figure 4B). Comparison of the two lineage gene sets identified a total of 5936 genes of which 3687 were shared by both groups. A further, 1213 genes were associated only with the *S. bockelmannii* group, including 43 cloud genes, whereas 1036 genes were restricted to the *S. sarumanii* group, including 15 cloud genes (Figure 4C). Overall, the pan-genome structure therefore separated the clinical isolates into two genomic lineages and showed a broader accessory-gene repertoire in the *S. bockelmannii* group. Given the limited number of isolates, genes found in only one group are more appropriately regarded as group-associated rather than species-specific.

### 3.9. Multi Locus Sequence Typing

Finally, multilocus sequence typing was performed using the allelic profiles of *adk*, *fumC*, *gyrB*, *icd*, *mdh*, and *recA*. The *S. bockelmannii* isolates Sm1, Sm2, Sm3, Sm5, Sm6, Sm7, and Sm8 were assigned to ST388 and shared the allelic profile *adk*-3, *fumC*-253, *gyrB*-191, *icd*-3, *mdh*-187, and *recA*-5 (Table 1). The *S. sarumanii* isolates Sm4, Sm9, and Sm10 showed the closest match to ST382, with the allelic profile *adk*-135, *fumC*-189, *icd*-161, *mdh*-131, and *recA*-147 (Table 1). However, the *gyrB* locus was not recovered from their genome assemblies, preventing definitive sequence-type assignment. These isolates were therefore classified as ST382-like. The MLST findings were consistent with the pan-genome analyses and further separated the clinical isolates into two distinct *Serratia* lineages. Core-genome phylogeny independently supported this separation, with the clinical isolates grouping according to their WGS-based species assignments and in relation to the corresponding reference genomes (Supplementary Figure S1).

## 4. Discussion and Conclusions

This study describes an outbreak of VIM-producing members of the *Serratia marcescens* complex among neonates admitted to the NICU of Santa Maria della Misericordia Hospital, Perugia. The investigation combined patient-level epidemiological data, routine microbiological surveillance, antimicrobial susceptibility testing, environmental and healthcare-worker screening, and genomic analysis. Eight neonates were colonized or infected with VIM-producing *Serratia*, during the November 2024 cluster, and five developed clinically apparent infection, including two bloodstream infections. Most affected neonates were first identified through rectal surveillance cultures, supporting gastrointestinal carriage as an important component of transmission in the neonatal setting. In three patients, colonization preceded subsequent clinical infection, illustrating the potential for progression from carriage to invasive disease in this vulnerable population. Similar observations have been reported in neonatal outbreaks, including one in which approximately one-quarter of colonized neonates subsequently developed infection, underscoring the value of active surveillance and early identification of colonized patients [50,51].

The temporal pattern of the outbreak was relatively concentrated. The first VIM-producing *Serratia* was identified on 6 November, followed by seven additional affected neonates over the next six days. After recognition of the cluster, colonized or infected neonates were transferred to isolation rooms or cohorted in a designated area of the NICU, and infection-control measures involving patient management, staff organization, and environmental surveillance were intensified. During December, VIM-producing *Serratia* continued to be recovered from surveillance cultures of neonates already involved in the outbreak, with the last positive detection recorded on 31 December 2024, but no new neonates with VIM-producing *Serratia* were identified after the November cluster. Thus, microbiological carriage persisted in previously affected patients, whereas no further incident cases were observed. The temporal association between implementation of infection-control measures and the absence of new cases is consistent with successful containment, although the retrospective nature of the investigation does not allow the contribution of any individual intervention to be determined.

The initial microbiological picture suggested dissemination of a VIM-producing *S. marcescens* strain, because routine identification classified the isolates as *S. marcescens* and the VIM-positive isolates showed closely similar multidrug-resistant phenotypes. WGS substantially changed this interpretation. The ten clinical isolates belonged to two species within the *S. marcescens* complex: seven isolates were assigned to *Serratia bockelmannii* ST388 and three to *Serratia sarumanii* ST382-like. Thus, the epidemiological cluster involved at least two distinct *Serratia* genomic backgrounds rather than a single clone. Pan-genome and core-genome analyses independently supported this separation, with *S. bockelmannii* showing a broader accessory-gene repertoire than *S. sarumanii*. Multiclonal *Serratia* outbreaks have also been reported in neonatal settings, where genomic analysis revealed co-circulating lineages that could not be reliably distinguished by routine susceptibility profiles alone [15,35,52].

The comparison between routine identification and WGS highlights the complementary clinical and epidemiological value of these approaches during outbreak investigation. MALDI-TOF MS provided rapid identification suitable for routine diagnostic management, but the isolates were initially reported as *S. marcescens* and the method did not resolve the distinction between *S. bockelmannii* and *S. sarumanii*. WGS subsequently demonstrated that apparently similar isolates belonged to two distinct species and genomic lineages, thereby changing the epidemiological interpretation from dissemination of a single *S. marcescens* strain to a more complex outbreak involving different genomic backgrounds. Beyond species identification, WGS also provided sequence-type assignment, resistance-gene profiles, plasmid replicon content, and information on specific resistance-gene variants. Thus, in this setting, WGS did not replace MALDI-TOF MS for rapid routine identification but provided the higher taxonomic and epidemiological resolution required to reconstruct the structure of the outbreak.

A similar distinction applied to carbapenemase detection. Rapid phenotypic and molecular testing provided timely identification of VIM production and therefore remained essential for immediate clinical and infection-control decisions. However, the routine molecular assay identifies a VIM-type carbapenemase without resolving the specific VIM allele. WGS showed that the clinical *Serratia*, *E. coli*, *C. freundii*, and *P. putida* isolates carried *bla*_VIM-1_, whereas the environmental *P. oleovorans* carried *bla*_VIM-2_. This distinction was epidemiologically relevant because it argued against a direct relationship between Po17 and the clinical *bla*_VIM-1_-positive cluster and represents information that could not be obtained from routine carbapenemase testing alone.

The resistance profile of the VIM-producing *Serratia* isolates has relevant therapeutic implications, particularly in neonates, for whom antimicrobial options are already limited. Resistance was observed to carbapenems and to β-lactam/β-lactamase inhibitor combinations including ceftazidime-avibactam and meropenem-vaborbactam consistent with the lack of inhibitory activity of avibactam and vaborbactam against metallo-β-lactamases [53]. Amikacin and fluoroquinolones retained in vitro activity, against the nine VIM-producing isolates, although therapeutic choices in neonates require consideration of age, infection site, pharmacokinetics, and toxicity.

Genomic analysis showed that carbapenem resistance formed part of a broader multidrug-resistance background. The VIM-producing *Serratia* isolates carried *bla*_VIM-1_ together with *bla*_SHV-12_, *sul*1, *dfr*A14, and several additional resistance genes. This indicates that carbapenem resistance occurred within a more complex multidrug-resistance background. Sm10 provides a useful contrast. Although it belonged to the same *S. sarumanii* ST382-like lineage as Sm4, it lacked these acquired determinants and showed a substantially more susceptible phenotype. This difference indicates that the multidrug-resistance phenotype was largely associated with acquisition of mobile resistance determinants rather than with species background alone. At the same time, the distribution of the intrinsic β-lactamase genes, with *bla*_SST-1_ in *S. bockelmannii* and *bla*_SRT-1_ in *S. sarumanii*, reflected the chromosomal background of the two lineages. A major finding of the genomic analysis was the reconstruction of the IncA-ST12 plasmid in the VIM-producing *Serratia* isolates. Illumina short-read sequencing identified a common IncA-ST12 replicon together with *bla*_VIM-1_ and *bla*_SHV-12_, but did not allow their physical co-localization to be established. Oxford Nanopore long-read sequencing of all nine VIM-producing *Serratia* isolates enabled reconstruction of complete circular IncA-ST12 plasmids in Sm1-Sm9 and direct localization of *bla*_VIM-1_ and *bla*_SHV-12_ on the same plasmid backbone, together with additional resistance determinants. The reconstructed plasmids showed 100% sequence coverage and 100% nucleotide identity across the nine isolates, supporting circulation of the same IncA-ST12 plasmid among the VIM-producing *Serratia* isolates.

The detection of *bla*_VIM-1_ in several bacterial species remains epidemiologically relevant. In addition to the two *Serratia* lineages, *bla*_VIM-1_ was detected in *E. coli*, *C. freundii*, and the clinical *P. putida* isolate Pp15. An IncA-ST12 replicon was also detected by Illumina sequencing in both *E. coli* isolates and in *C. freundii* Cf13. Because IncA plasmids are conjugative and have a broad host range, the shared replicon profile may reflect circulation of a related plasmid backbone among different *Enterobacterales* [27,54,55,56]. However, long-read sequencing was not performed for the *E. coli* or *C. freundii* isolates. Consequently, physical co-localization of *bla*_VIM-1_, *bla*_SHV-12_, and the IncA-ST12 replicon has been demonstrated directly only in the *Serratia* isolates, and horizontal transfer of the same plasmid between bacterial species cannot be concluded from the present data. Similarly, no plasmid replicon was detected in clinical *P. putida* Pp15, and the genomic location of *bla*_VIM-1_ in this isolate remains unresolved. The IncM1 replicon was restricted to the *S. bockelmannii* isolates and was absent from the *S. sarumanii* group, providing an additional genomic marker separating the two lineages. Other plasmid families were detected in the *E. coli* and *C. freundii* isolates, further illustrating the diversity of mobile elements present during the outbreak.

Environmental and healthcare-worker screening did not identify a reservoir of the outbreak-associated *Serratia* lineages. Environmental sampling began the day after the first VIM-producing *Serratia* case and included repeated sampling of high-touch surfaces, water samples from seven taps, and hand screening of healthcare workers. No *Serratia* was recovered from these samples. The recovery of VIM-producing *P. oleovorans* Po17 from a sink trap nevertheless showed that VIM-producing Gram-negative bacteria were present in the water-associated environment. WGS demonstrated that Po17 carried chromosomal *bla*_VIM-2_ rather than *bla*_VIM-1_, and no shared plasmid replicon with the clinical isolates was identified. These findings argue against Po17 as the direct source of the clinical *bla*_VIM-1_ cluster and instead indicate an independent environmental occurrence of VIM-mediated carbapenem resistance. The absence of an identified environmental source does not exclude environmental transmission. Sampling was performed after recognition of the outbreak and at defined time points, and intermittent or low-level contamination may therefore have escaped detection. Patient-to-patient transmission through shared care activities also cannot be reconstructed completely. Although the NICU layout and cohorting procedures were documented, detailed retrospective information on the exact position and proximity of individual affected neonates was not available. The study was retrospective, and genomic analysis was restricted to isolates that had been stored and remained available. These limitations should be considered when interpreting transmission pathways. The increased microbiological surveillance during November and December also needs to be considered when interpreting the outbreak chronology. No VIM-producing *Serratia* had been identified before November, despite routine screening, while detection of the first case prompted intensified surveillance and outbreak-control activities. The absence of new affected neonates after the initial November cluster, despite continued and substantially increased sampling, supports the observation that no further incident VIM-producing *Serratia* cases occurred during follow-up. However, the data do not allow a single intervention to be identified as responsible for containment.

Overall, the integration of epidemiological and genomic data substantially changed the interpretation of this outbreak. What initially appeared by routine microbiology to represent dissemination of VIM-producing *S. marcescens* was resolved into the circulation of two species within the *S. marcescens* complex, together with *bla*_VIM-1_-positive isolates belonging to other Gram-negative species. Rapid MALDI-TOF MS identification and molecular carbapenemase testing remained essential for timely clinical management and infection-control decisions, whereas WGS provided the additional resolution required to distinguish co-circulating bacterial lineages, discriminate *bla*_VIM_ alleles, and characterize the mobile resistance and plasmid backgrounds of the isolates. Long-read sequencing further extended this information by demonstrating circulation of an identical IncA-ST12 plasmid among the VIM-producing *Serratia* isolates. These findings illustrate the complementary role of rapid routine diagnostics and genomic epidemiology in the investigation of complex healthcare-associated outbreaks.

## Figures and Tables

**Table 1:** Demographic, clinical and microbiological characteristics of the clinical and environmental isolates selected for genomic analysis during the investigation of a *Serratia* outbreak.

Isolate ID	Species by WGS (ST)	Patient	Gestational Age (Weeks)	Sex	Sampling Date (2024)	Days from Admission to Sampling	Clinical STATUS	*bla*_VIM_ Status
Sm1	*Serratia bockelmannii* (ST388)	A	29	F	06/11	17	Ocular infection	Positive
Sm2	*Serratia bockelmannii* (ST388)	B	30	F	10/11	18	bloodstream infection	Positive
Sm3	*Serratia bockelmannii* (ST388)	C	27	M	11/11	3	Rectal colonization	Positive
Sm4	*Serratia sarumanii* (ST382-like)	D	29	F	11/11	11	Rectal colonization	Positive
Sm5	*Serratia bockelmannii* (ST388)	E	24	F	11/11	56	Rectal colonization	Positive
Sm6	*Serratia bockelmannii* (ST388)	F	40	M	12/11	0	Rectal colonization	Positive
Sm7	*Serratia bockelmannii* (ST388)	G	34	F	12/11	6	Rectal colonization	Positive
Sm8	*Serratia bockelmannii* (ST388)	H	40	M	12/11	2	Rectal colonization	Positive
Sm9	*Serratia sarumanii* (ST382-like)	D	29	F	19/11	19	bloodstream infection	Positive
Sm10	*Serratia sarumanii* (ST382-like)	I	24	F	17/11	85	bloodstream infection	Negative
Ec12	*Escherichia coli*	E	16/09	F	14/11	59	Rectal colonization	Positive
Cf13	*Citrobacter freundii*	H	10/11	M	17/11	7	Rectal colonization	Positive
Ec14	*Escherichia coli*	D	31/10	F	15/11	15	Rectal colonization	Positive
Pp15	*Pseudomonas putida*	L	12/09	F	21/11	69	Rectal colonization	Positive
Pp16	*Pseudomonas putida*	-	-	-	13/11	-	Sink swab	Negative
Po17	*Pseudomonas oleovorans*	-	-	-	13/11	-	Sink swab	Positive

ST, sequence type; *bla*_VIM_: Verona integron-encoded metallo-β-lactamase gene; F, female; M, male.

## Data Availability

The bacterial genome sequencing data generated and analysed during the present study have been deposited in the National Center for Biotechnology Information Se-quence Read Archive (SRA) under BioProject accession n° PRJNA1498554. Further inquiries can be directed to the corresponding authors.

## Supplementary Materials

The following supporting information can be downloaded at: https://www.mdpi.com/article/10.3390/pathogens15101033/s1, Table S1: The complete list of 56 high-touch surfaces samples and location whitin the NICU; Table S2: The complete list of 14 water samplings and location within the NICU; Table S3: Minimum inhibitory concentration (MIC) values for the antimicrobial agents tested against the *Serratia* isolates. MIC values are expressed in mg/L; Table S4: Detailed Illumina sequencing and assembly quality metrics for the *Serratia* isolates, including the numbers of raw and quality-filtered reads; Table S5: Detailed Nanopore sequencing output and quality metrics for the *Serratia* isolates, including total bases generated, percentage of passed bases, total number of reads, and percentage of passed reads; Figure S1: Core-genome phylogeny of the *Serratia* isolates. Core-genome phylogenetic tree including the 10 *Serratia* isolates analysed in this study and the reference genomes *S. marcescens* ELP1.10, *S. marcescens* subsp. marcescens Db11, *S. sarumanii* K-M0228, and *S. bockelmannii* KL0361. Branches are coloured according to species assignment, with *S. sarumanii* shown in green and *S. bockelmannii* in orange. The scale bar indicates the number of substitutions per site.
