# Supplementary material for: Whole-Genome Sequencing Identified Co-Circulating VIM-Producing Serratia bockelmannii and Serratia sarumanii in a Neonatal Intensive Care Unit Cluster Investigation
Source: Pathogens. 2026 Oct 1;15(10):1033. doi: 10.3390/pathogens15101033 (PMC13708157; doi:10.3390/pathogens15101033)
Supplement: Supplementary file 1 [file pathogens-15-01033-s001.zip › pathogens-4527118-supplementary.pdf]

Supplementary materials

Supplementary Table S1. The complete list of 56 high-touch surfaces samples and location within the NICU

Intermediate Care Unit	Intensive Care Room	Corridor	Isolation room
Stainless-steel sink siphon	Hand gel at the entrance	Ultrasound machine 1– probes + ultrasound gel	Infant Scale
Plastic sink siphon	Infant scale	Ultrasound machine 1– keyboard	Vaseline jarno. 3
Diaper cream no. 1 and diaper cream no. 2	White sink siphon	Ultrasound machine 2– keyboard + probe + gel	Sink siphon
Changing table near the sink	Diaper scale	Ultrasound machine 3 – keyboard + probes + gel	
Dermolan cream on the changing table	Vaseline jar no. 2	Cabinet	
CICABEN cream on the changing table	Therapy computer keyboard		
Microderma *cream on the changing table*	Large Vaseline jar		
Vaseline no. 1	Entrance to Microprem Room		
Keyboard and mouse of INTEL PC – therapy unit AB0048414	PC keyboard		
Entrance changing-table surface	Back of the Microprem room		
Sideral *and* Entero 4	Disinfectant bottle		
Green cabinet – rectal probes	Yellow-green-blue probes cabinet		
Blue cabinet – gastric probes	Red cabinet – rectal probes		
Red cabinet – probes near entrance changing table	Adhesive tape		
Ochre-yellow cabinet AM0033551	Vaseline in tube (cream formulation)		
Ochre cabinet AM0033660	Thermometer		
Vaseline no. 1	Incubator 1– water filter		
Adhesive tape	Incubator 1– air filter		

Vaseline from the corridor	Incubator 2 – water filter		
Thermometer behind crib 01319	Incubator 2 – air filter		
Thermometer behind crib 01329	Bathroom door handle (inside)		
Feeding bottle warmer 1	Bathroom door handle (outside)		
Feeding bottle warmer 2	Micro CL centrifuge		
Feeding bottle warmer 3	Blood gas analyzer Radiometer ABL 90 Flex		

Supplementary Table S2. The complete list of 14 water samplings and location within the NICU

Intermediate Care Unit	Intensive Care Room	Isolation rooms
Sink faucet no. 1 near door p+2 035 without filter	Sink faucet No. 4 P+2 032<br>With filter	sink faucet no. 6near door 029<br>without filter
Sink faucet no. 2 near door p+2 033 without filter	Sink faucet no. 5 between the columns<br>without filter	Sink faucet no. 7<br>with filter
Sink faucet no. 13 near windows with filter		

Supplementary Table S3. Minimum inhibitory concentration (MIC) values for the antimicrobial agents tested against the *Serratia* isolates. MIC values are expressed in mg/L.

**Antibiotic tested**	**Sample ID**									
	Sm1	Sm2	Sm3	Sm4	Sm5	Sm6	Sm7	Sm8	Sm9	Sm10
Cefotaxime	>2	>2	>2	>2	>2	>2	>2	>2	>2	>2
Ceftazidime	>32	>32	>32	>32	>32	>32	>32	>32	>32	<=0,12
Ceftazidime-Avibactam	16	16	64	16	64	16	16	16	16	<=0.12
Ceftolozane-Tazobactam	8	8	64	64	64	8	8	8	8	16
Imipenem	8	8	8	8	8	8	8	8	8	1
Meropenem	16	16	16	16	32	16	16	16	16	≤0,25
Meropenem-Vaborbactam	32	32	32	32	32	32	32	32	32	0
Trimetoprim-Sulfametazole	4	4	8	8	8	4	4	4	4	0
Ciprofloxacin	0,25	0,25	0,25	0,25	0,25	0,25	0,25	0,25	0,25	0,25
Levofloxacin	0,5	0,5	0,5	0,5	0,5	0,5	0,5	0,5	0,5	0,5
Amikacin	4	4	4	4	4	4	4	4	4	4

Supplementary Table S4: Detailed Illumina sequencing and assembly quality metrics for the *Serratia* isolates, including the numbers of raw and quality-filtered reads.

Run	Samples ID	N. of total reads	N. of total passing filter reads	% Reads passing filter	Assembly lenght	Number of contigs	N50	GC content	Sequencing depth
1		35.887.816	31.214.514	87,0					
	Sm1		2.926.977		5.412.071	36	347.272	59	138
	Sm2		3.727.294		5.411.225	31	347.272	59	176
	Sm3		2.622.037		5.413.005	35	355.746	59	122
	Sm4		2.951.123		5.158.661	32	580.228	59	147
	Sm5		3.010.451		5.411.444	35	347.084	59	141
	Sm6		3.875.458		5.409.872	35	347.142	59	180
	Sm7		2.860.627		5.411.732	30	599.389	59	135
	Sm8		2.686.069		5.409.723	32	347.272	58	128
	Sm9		3.079.638		5.158.838	33	518.709	59	152
	Sm10		3.474.901		5.009.642	24	580.492	60	177
2		40.925.790	37.878.686	92,6					
	Ec12		6.758.282		5.304.397	74	342.142	50	174
	Cf13		6.554.326		5.573.484	94	220.506	51	160
	Ec14		6.456.293		5.303.391	75	326.027	50	164
	Pp15		6.798.772		6.269.347	96	179.106	61	148
	Pp16		6.463.114		6.378.959	76	232.691	61	137
	Po17		4.847.564		4.599.379	110	93.986	62	144

Supplementary Table S5. Detailed Nanopore sequencing output and quality metrics for the *Serratia* isolates, including total bases generated, percentage of passed bases, total number of reads, and percentage of passed reads.

Samples	Total bases (Mb)	Passed bases	Total reads (k)	Passed reads (%)
Sm1	202.15	96.10	83.99	85.60
Sm2	684.26	97.00	199.26	88.90
Sm3	354.59	97.30	101.83	90.20
Sm4	307.56	95.20	128.28	83.50
Sm5	560.20	97.40	138.90	91.90
Sm6	267.84	96.20	102.45	87.10
Sm7	405.59	95.90	187.61	85.90
Sm8	152.78	94.80	71.84	84.60
Sm9	440.55	96.40	171.98	86.90

Supplementary Figure S1

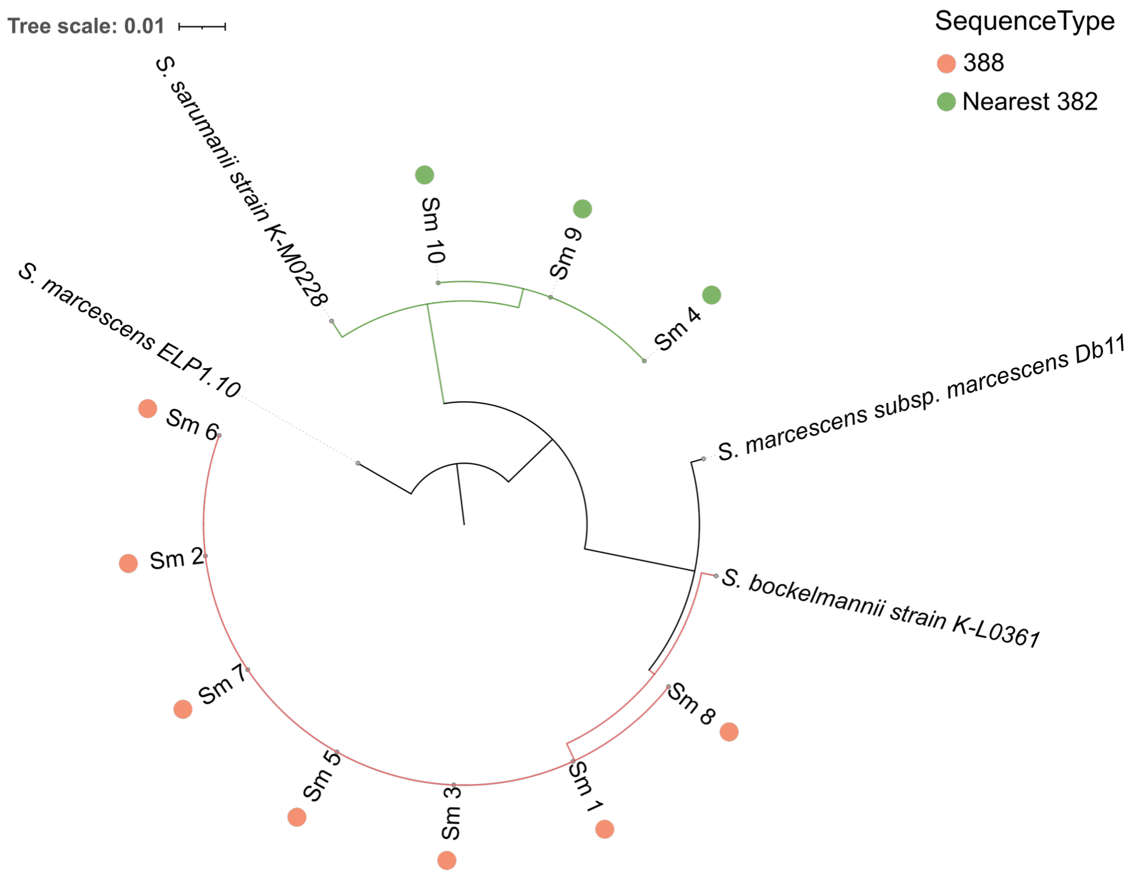

Figure S1. Core-genome phylogeny of the *Serratia* isolates. Core-genome phylogenetic tree including the 10 *Serratia* isolates analysed in this study and the reference genomes *S. marcescens* ELP1.10, *S. marcescens* subsp. *marcescens* Db11, *S. sarumanii* K-M0228, and *S. bockelmannii* K-L0361. Branches are coloured according to species assignment, with *S. sarumanii* shown in green and *S. bockelmannii* in orange. The scale bar indicates the number of substitutions per site.
